# Supplementary material for: Multi-Fold Computational Analysis to Discover Novel Putative Inhibitors of Isethionate Sulfite-Lyase (Isla) from Bilophila wadsworthia: Combating Colorectal Cancer and Inflammatory Bowel Diseases
Source: Cancers (Basel). 2023 Jan 31;15(3):901. doi: 10.3390/cancers15030901 (PMC9913583; doi:10.3390/cancers15030901)
Supplement: Supplementary file 1 [file cancers-15-00901-s001.zip › cancers-2146082-supplementary.pdf]

Supporting Information
Multi-fold Computational Analysis to Discover Novel Putative Inhibitors of isethionate sulfite-lyase (Isla) from *Bilophila wadsworthia:* Combating colorectal cancer and inflammatory bowel diseases

Muhammad Waqas[1,2†], Sobia Ahsan Halim[1†], Atta Ullah[1,3], Assim Alaa Mohammed Ali[1], Asaad Khalid[4,5], Ashraf N. Abdalla[6], Ajmal Khan[1*], Ahmed Al-Harrasi[1,*]

1 Natural and Medical Sciences Research Center, University of Nizwa, P.O Box 33, Postal Code 616, Birkat Al Mauz, Nizwa, Sultanate of Oman
2 Department of Biotechnology and genetic Engineering, Hazara University Mansehra, Mansehra 2100, Pakistan
3 Department of Zoology, Abdul Wali Khan University, Mardan 23200, Pakistan
4 Substance Abuse and Toxicology Research Center, Jazan University, P.O. Box: 114, Jazan 45142, Saudi Arabia
5 Medicinal and Aromatic Plants and Traditional Medicine Research Institute, National Center for Research, P. O. Box 2404, Khartoum, Sudan
6 Department of Pharmacology and Toxicology, College of Pharmacy, Umm Al-Qura University, Makkah 21955, Saudi Arabia
* *Correspondence: ajmalkhan@unizwa.edu.om (A.K); aharrasi@unizwa.edu.om (A.A-H)
† These Authors contributes equally

Table of Contents

Table S1 Physiochemical properties of selected inhibitors from SwissADME server.
Table S2 Pharmacokinetics properties of selected inhibitors from SwissADME server
Table S3 Drug likeness and Medicinal chemistry properties of selected inhibitors from the SwissADME server.
Table S4 Detailed hydrogen bond interactions of Isla protein with the selected inhibitors and reference agonist attached (7KQ3) complexes versus simulation time.
Figure S1 Root mean square histogram analysis of Isla in free state (7KQ4), reference substrate attached (7KQ3) and in complex with selected putative inhibitors.
Figure S2. The covariance matrix plotted against the corresponding eigenvector from each system to obtain the first ten eigenvectors in fraction. The eigenvectors contribution for each system is reported in percentage.

Table S1. Physiochemical properties of compounds **1-9** calculated by SwissADME server.

Compounds	ZINC ID	Physiochemical properties						Water Solubility	Lipophilicity	Drug likeness		
		MWT (g/mol)	TPSA (A^2)	NRB	MR	HBA	HBD	Class	Log Po/w	Lipinski	Veber	Muegge
CP1	Fc1cnc(c(n1)C(=O)N)O	157.1	89.1	1	32.11	5	2	Very soluble	0.7	0	0	1
CP2	Nn1c(=S)[nH]nc1c1ccncc1	193.23	104.61	1	50.79	2	2	Very soluble	0.88	0	0	1
CP3	OC(=O)CCc1ccc(cc1)O	166.17	57.53	3	44.82	3	2	Very soluble	1.19	0	0	1
CP4	Nn1c(=S)[nH]nc1c1cccnc1	193.23	104.61	1	50.79	2	2	Very soluble	0.97	0	0	1
CP5	Sc1nnc(o1)c1cc2c([nH]1)cccc2	217.25	93.51	1	58.84	3	1	Soluble	2.11	0	0	0
CP6	Clc1ccc(c(c1)F)c1nnc(o1)S	230.65	77.72	1	51.95	4	0	Soluble	2.32	0	0	0
CP7	Sc1nnc(o1)Cc1ccccc1F	210.23	77.72	2	50.96	4	0	Soluble	2.2	0	0	0
CP8	NC(=S)N/N=C/c1ccc(cc1)[N+](=O)[O-]	224.24	128.32	4	62.02	3	2	Soluble	1.36	0	0	0
CP9	CCNC(=S)N/N=C(/c1cc(sc1C)C)\C	255.4	96.75	5	75.52	1	2	Soluble	3.1	0	0	0

MWT = Molecular Weight, TPSA = Topological Polar Surface Area, NRB = Number of Rotatable bond, MR = Molar Refractivity, HBA = Hydrogen bond acceptor, HBD = Hydrogen bond donor, Log Po/w = Partition Coefficient octanol/water

MWT = Molecular weight , TPSA = topological polar surface area, NRB = Number of rotatable bonds, MR = Molar refractivity, HBA = Hydrogen bond acceptor, HBD = hydrogen bond donor, Log Po/w = Partition coefficient octanol/water.

Table S2. Pharmacokinetics properties of compounds **1-9** calculated by SwissADME server.

**Compounds**	**SMILE**	**Pharmacokinetics**									
			**GIA**	**BBBP**	**P-gp S**	**CYP 1A2 inhibitor**	**CYP 2C19 inhibitor**	**CYP 2C9 inhibitor**	**CYP 2D6 inhibitor**	**CYP 3A4 inhibitor**	**Log Kp**
CP1	Fc1cnc(c(n1)C(=O)N)O		High	No	No	No	No	No	No	No	-7.29
CP2	Nn1c(=S)[nH]nc1c1ccncc1		High	No	No	No	No	No	No	No	-7.61
CP3	OC(=O)CCc1ccc(cc1)O		High	Yes	No	No	No	No	No	No	-6.49
CP4	Nn1c(=S)[nH]nc1c1cccnc1		High	No	No	No	No	No	No	No	-7.61
CP5	Sc1nnc(o1)c1cc2c([nH]1)cccc2		High	No	No	Yes	No	No	No	Yes	-6.16
CP6	Clc1ccc(c(c1)F)c1nnc(o1)S		High	Yes	No	Yes	No	No	No	No	-5.85
CP7	Sc1nnc(o1)Cc1ccccc1F		High	Yes	No	Yes	No	No	No	No	-5.96
CP8	NC(=S)N/N=C/c1ccc(cc1)[N+](=O)[O-]		High	No	No	Yes	No	No	No	No	-6.69
CP9	CCNC(=S)N/N=C(/c1cc(sc1C)C)\C		High	No	No	Yes	Yes	Yes	No	No	-5.96

GIA = gastrointestinal absorption, BBBP = Blood brain barrier permeability, P-gp S = P-glycoprotein substrate, Log Kp = skin permeation.

Table S3. Drug likeness and medicinal chemistry properties of compounds **1-9** estimated by SwissADME server.

Compounds	SMILE	Drug likeness	Medicinal chemistry		
		Bio Availability Score	PAINS Alert	Lead likeness	Synthetic accessibility
CP1	Fc1cnc(c(n1)C(=O)N)O	0.55	0	1	2.03
CP2	Nn1c(=S)[nH]nc1c1ccncc1	0.55	0	1	2.21
CP3	OC(=O)CCc1ccc(cc1)O	0.85	0	1	1
CP4	Nn1c(=S)[nH]nc1c1cccnc1	0.55	0	1	2.34
CP5	Sc1nnc(o1)c1cc2c([nH]1)cccc2	0.55	0	1	2.13
CP6	Clc1ccc(c(c1)F)c1nnc(o1)S	0.55	0	1	2.12
CP7	Sc1nnc(o1)Cc1ccccc1F	0.55	0	1	2.02
CP8	NC(=S)N/N=C/c1ccc(cc1)[N+](=O)[O-]	0.55	0	1	2.28
CP9	CCNC(=S)N/N=C(/c1cc(sc1C)C)\C	0.55	0	0	3.09

Table S4. Hydrogen bond interactions of Isla with the selected ligands and reference (7KQ3) versus simulation time.

Compound	H-Acceptor	H-Donor	Bond life (%)	Avg-Distance (Å)	Avg-Angle°
7KQ3	GLU470-OE2	8X3831-O6	52.43%	2.71	163.76
	8X3831-O4	ARG678-NH1	42.34%	2.81	160.92
	8X3831-O5	ARG189-NH2	40.67%	2.82	153.83
	8X3831-O7	TYR485-OH	36.45%	2.73	161.65
	8X3831-O5	ARG189-NH1	34.26%	2.81	153.67
	8X3831-O7	ARG189-NH1	28.00%	2.81	156.12
	8X3831-O5	ARG678-NH1	24.50%	2.81	159.18
	8X3831-O5	TYR485-OH	23.98%	2.74	161.77
	8X3831-O4	TYR485-OH	22.53%	2.74	161.51
	8X3831-O4	ARG189-NH1	22.47%	2.78	155.23
	8X3831-O4	TYR587-OH	22.26%	2.71	161.09
	8X3831-O7	TYR587-OH	22.08%	2.69	161.89
	8X3831-O5	TYR587-OH	21.79%	2.71	160.30
	8X3831-O7	ARG189-NH2	20.22%	2.83	152.06
	8X3831-O7	ARG678-NH1	20.16%	2.81	160.04
	8X3831-O7	ARG678-NH2	19.09%	2.86	160.81
	8X3831-O4	ARG189-NH2	19.05%	2.83	150.74
	8X3831-O6	GLN193-NE2	16.70%	2.90	159.33
	8X3831-O6	CYS468-N	15.89%	2.88	158.70
	8X3831-O4	ARG678-NH2	8.40%	2.86	157.03
	8X3831-O5	ARG678-NH2	6.80%	2.85	153.83
	8X3831-O7	THR312-OG1	6.44%	2.77	157.18
	THR312-OG1	8X3831-O6	6.24%	2.84	159.78
	GLU470-OE1	8X3831-O6	1.24%	2.79	162.82
	8X3831-O7	GLN193-NE2	0.54%	2.85	166.16
	8X3831-O6	THR312-OG1	0.45%	2.83	164.88
	VAL680-O	8X3831-O6	0.17%	2.82	156.84
	8X3831-O5	THR312-OG1	0.09%	2.72	158.50
	TRP374-NE1	8X3831-O6	0.07%	2.93	146.89
	8X3831-O6	TYR587-OH	0.02%	2.88	152.78
	8X3831-O5	TRP374-NE1	0.01%	2.86	139.43
	8X3831-O6	GLN193-N	0.01%	2.91	140.64
	8X3831-O6	ARG678-NH1	0.01%	2.92	142.68
	8X3831-O4	GLY483-N	0.01%	2.93	163.93
	8X3831-O7	TRP374-NE1	0.01%	2.90	149.77
	8X3831-O6	TRP374-NE1	0.01%	2.96	144.22
CP1	CP1831-O7	ARG189-NH1	60.24%	2.82	153.43
	THR312-OG1	CP1831-N6	52.20%	2.87	158.11
	CP1831-O10	TYR587-OH	44.53%	2.78	160.36
	CP1831-O7	GLN193-NE2	34.99%	2.85	157.99
	CP1831-O10	ARG189-NH2	32.87%	2.86	155.73
	GLU589-OE1	CP1831-N9	22.41%	2.83	153.98
	GLU589-OE2	CP1831-N9	22.03%	2.82	158.68
	CP1831-O10	ARG189-NH1	13.54%	2.87	150.30
	GLY311-O	CP1831-N6	5.58%	2.89	157.98
	TYR485-OH	CP1831-N9	5.22%	2.90	144.39
	CP1831-O7	GLN193-N	3.93%	2.92	153.09
	CP1831-F11	PHE682-N	1.38%	2.89	140.97
	VAL680-O	CP1831-N9	1.33%	2.85	143.80
	TYR485-OH	CP1831-N9	0.55%	2.85	151.43
	CP1831-N9	TYR485-OH	0.48%	2.89	144.91
	CP1831-O7	ARG189-NH2	0.31%	2.91	145.44
	CP1831-O10	ARG678-NH2	0.20%	2.88	148.13
	GLU589-OE2	CP1831-N9	0.19%	2.87	145.00
	GLU589-OE1	CP1831-N9	0.17%	2.84	145.44
	CP1831-N9	ARG678-NH2	0.10%	2.90	141.91
	CP1831-N3	ARG678-NH2	0.09%	2.89	141.50
	CP1831-O10	GLN193-NE2	0.08%	2.93	139.33
	ARG678-NH2	CP1831-N9	0.05%	2.90	137.91
	CP1831-N9	ARG678-NH2	0.03%	2.96	139.20
CP2	TYR479-OH	CP2831-N7	78.58%	2.80	152.16

	ASN675-OD1	CP2831-N13	75.73%	2.72	145.32
	CP2831-O11	ALA484-N	21.40%	2.90	156.83
	CP2831-N14	TYR587-N	0.14%	2.96	144.17
	GLN544-OE1	CP2831-N13	0.13%	2.70	144.49
	TYR479-OH	CP2831-N13	0.01%	2.82	139.66
	GLY482-O	CP2831-N7	0.01%	2.83	138.53
CP4	TYR587-O	CP4831-N14	61.47%	2.85	159.09
	SER481-O	CP4831-N11	15.32%	2.91	152.38
	TYR479-OH	CP4831-N11	4.11%	2.90	151.32
	GLN544-OE1	CP4831-N14	3.00%	2.87	147.99
	CP4831-N11	GLY483-N	2.10%	2.94	145.89
	CP4831-N3	GLY483-N	1.06%	2.95	155.68
	CP4831-N14	TYR587-N	0.34%	2.96	145.76
	CP4831-N3	THR185-OG1	0.01%	2.97	162.45
CP5	GLU589-OE1	CP5831-N4	96.38%	2.75	162.09
	GLU589-OE1	CP5831-N7	37.20%	2.87	147.74
	CP5831-O15	ARG189-NH1	16.20%	2.88	152.27
	CP5831-O14	ARG189-NH1	14.16%	2.88	152.54
	CP5831-N3	TYR485-OH	14.11%	2.87	162.63
	TYR587-O	CP5831-N7	5.47%	2.88	151.08
	ALA484-O	CP5831-N7	4.24%	2.89	143.61
	CP5831-N4	TYR485-OH	1.01%	2.93	145.22
	CP5831-O15	ARG678-NH2	0.95%	2.90	147.57
	CP5831-O14	ARG678-NH2	0.88%	2.91	149.07
	CP5831-N3	TYR587-OH	0.86%	2.89	157.04
	GLY483-O	CP5831-N7	0.61%	2.91	160.82
	CP5831-N3	ARG189-NH2	0.58%	2.93	156.35
	CP5831-O14	ARG678-NH1	0.40%	2.90	146.48
	CP5831-O15	ARG678-NH1	0.35%	2.90	148.07
	CP5831-O14	ARG189-NH2	0.31%	2.91	145.85
	CP5831-O15	ARG189-NH2	0.28%	2.91	145.24
	CP5831-O15	CYS468-N	0.24%	2.90	147.41
	CP5831-O15	TRP374-NE1	0.15%	2.90	143.00
	CP5831-O14	TRP374-NE1	0.14%	2.91	141.38
	CP5831-N13	ARG189-NH1	0.13%	2.96	148.60
	CP5831-O15	TYR485-OH	0.07%	2.85	147.35
	CP5831-O14	TYR485-OH	0.04%	2.89	143.26
	CP5831-O14	ARG189-NH1	0.02%	2.85	143.57
	ALA484-O	CP5831-N7	0.02%	2.88	151.60
	CP5831-O15	THR312-OG1	0.01%	2.76	140.87
	CP5831-O15	ARG189-NH1	0.01%	2.84	144.17
	CP5831-O14	THR312-OG1	0.01%	2.95	148.11
CP8	GLU589-OE1	CP8831-N12	63.64%	2.80	162.38
	GLY483-O	CP8831-N10	39.57%	2.79	150.80
	CP8831-N2	GLN193-NE2	24.07%	2.92	166.19
	GLU589-OE1	CP8831-N12	23.22%	2.82	158.58
	TYR587-O	CP8831-N12	10.00%	2.90	158.15
	CP8831-N11	ARG189-NH2	9.99%	2.92	151.07
	CP8831-N11	THR185-OG1	2.36%	2.88	150.28
	TYR587-O	CP8831-N12	1.91%	2.91	158.33
	THR185-OG1	CP8831-N10	0.53%	2.84	148.48
	CP8831-N2	GLN193-N	0.52%	2.96	154.19
	CP8831-N11	ARG189-NE	0.15%	2.94	149.16
	TYR479-OH	CP8831-N12	0.11%	2.93	153.40
	TYR485-OH	CP8831-N12	0.09%	2.90	155.88
	GLU470-OE2	CP8831-N12	0.07%	2.85	148.78
	GLU470-OE2	CP8831-N12	0.06%	2.83	151.69
	CP8831-N11	TYR485-OH	0.06%	2.94	152.14
	TYR485-OH	CP8831-N10	0.01%	2.96	148.40
	TYR479-OH	CP8831-N12	0.01%	2.93	141.07
CP9	CP9831-N11	THR185-OG1	60.24%	2.80	162.23
	GLY483-O	CP9831-N10	59.63%	2.82	160.41
	GLN193-OE1	CP9831-N12	18.96%	2.85	152.76
	SER481-O	CP9831-N12	17.22%	2.85	163.24
	GLU470-OE2	CP9831-N12	12.60%	2.85	150.52
	CP9831-N3	ARG189-NH2	12.57%	2.90	150.03

	GLN193-OE1	CP9831-N12	12.14%	2.85	152.90
	GLU470-OE2	CP9831-N12	9.02%	2.84	152.17
	ALA484-O	CP9831-N10	5.47%	2.88	148.73
	TYR587-O	CP9831-N10	4.03%	2.80	141.43
	SER481-O	CP9831-N12	2.13%	2.87	163.31
	CP9831-N12	GLN193-NE2	1.07%	2.92	147.04
	CP9831-N10	THR185-OG1	0.52%	2.95	144.10
	CP9831-N3	ARG189-NE	0.45%	2.95	144.30
	CP9831-N3	TYR485-OH	0.14%	2.84	159.58
	GLY483-N	CP9831-N10	0.09%	2.95	140.27
	CP9831-N12	ARG678-NH1	0.09%	2.94	149.87
	CP9831-N11	TYR485-OH	0.08%	2.86	161.44
	TYR587-OH	CP9831-N12	0.07%	2.94	152.90
	ARG678-NH2	CP9831-N12	0.06%	2.93	153.20
	CP9831-N3	ARG189-NH2	0.05%	2.88	145.30
	TYR485-OH	CP9831-N12	0.05%	2.95	155.91
	ARG678-NH1	CP9831-N12	0.04%	2.89	148.34
	CP9831-N3	ARG189-NH1	0.04%	2.90	152.50
	CP9831-N11	GLN193-NE2	0.04%	2.90	165.08
	GLY483-O	CP9831-N12	0.04%	2.92	160.74
	TYR485-OH	CP9831-N12	0.04%	2.95	152.67
	ARG678-NH1	CP9831-N12	0.04%	2.95	153.42
	GLN544-OE1	CP9831-N10	0.03%	2.91	148.93
	ARG678-NH2	CP9831-N12	0.02%	2.97	146.71
	TYR479-OH	CP9831-N12	0.01%	2.95	154.22
	GLN193-NE2	CP9831-N12	0.01%	2.94	150.60

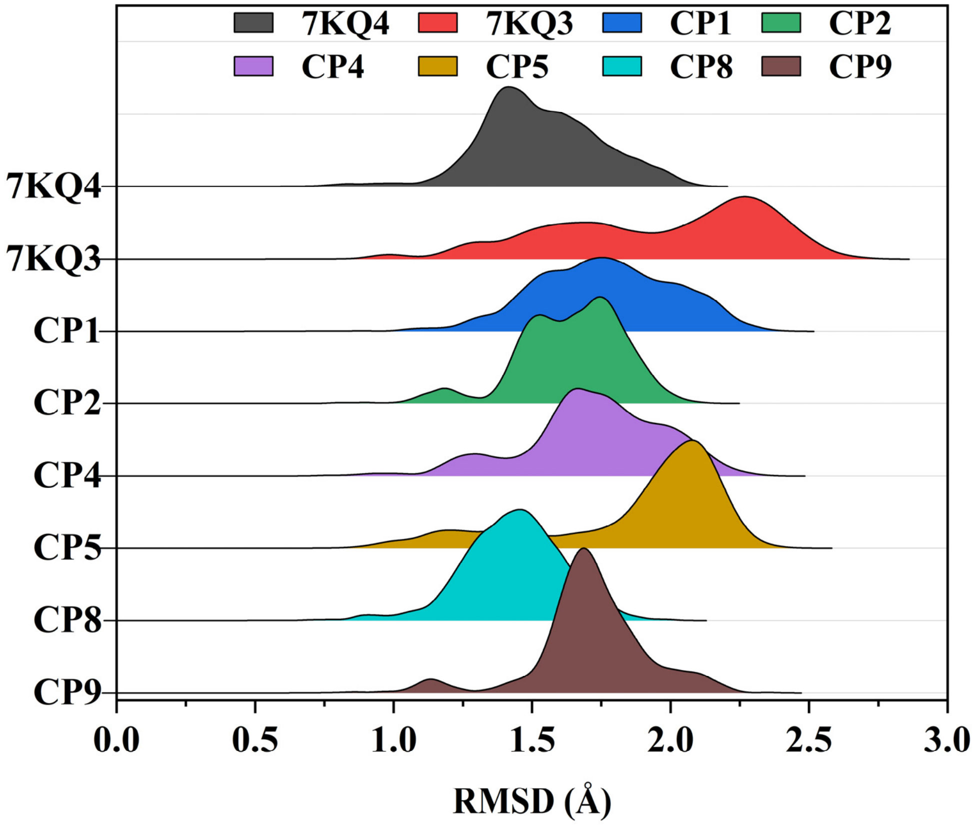

Figure S1 Root mean square histogram analysis of Isla in free state (7KQ4), reference substrate attached (7KQ3) and in complex with selected putative inhibitors.

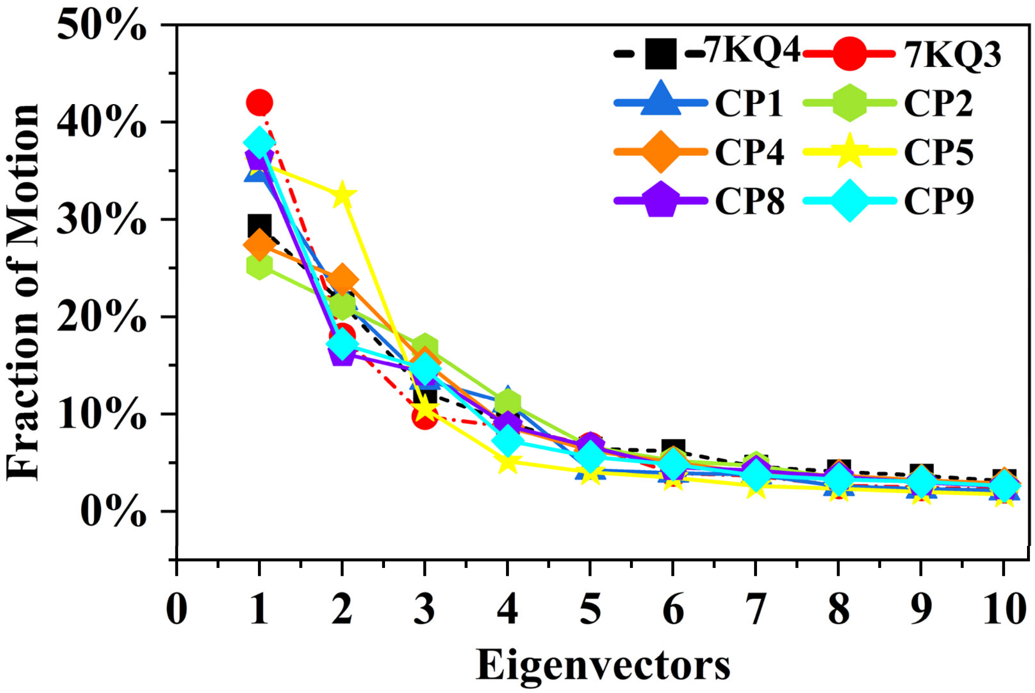

Figure S2. The covariance matrix plotted against the corresponding eigenvector from each system to obtain the first ten eigenvectors in fraction. The eigenvectors contribution for each system is reported in percentage.
